# The dose-response effects of flurbiprofen, indomethacin, ibuprofen, and naproxen on primary skeletal muscle cells

**DOI:** 10.1080/15502783.2024.2302046

**Published:** 2024-01-10

**Authors:** Brandon M. Roberts, Alyssa V. Geddis, Ronald W. Matheny

**Affiliations:** a Military Performance Division, US Army Research Institute of Environmental Medicine, Natick, MA, USA; b Military Operational Medicine Research Program, Detrick, MD, USA

**Keywords:** Myogenesis, anabolic signaling, skeletal muscle, AKT, muscle growth

## Abstract

**Background:**

Non-steroidal anti-inflammatory drugs (NSAIDs) like ibuprofen, flurbiprofen, naproxen sodium, and indomethacin are commonly employed for their pain-relieving and inflammation-reducing qualities. NSAIDs work by blocking COX-1 and/or COX-2, enzymes which play roles in inflammation, fever, and pain. The main difference among NSAIDs lies in their affinity to these enzymes, which in turn, influences prostaglandin secretion, and skeletal muscle growth and regeneration. The current study investigated the effects of NSAIDs on human skeletal muscle cells, focusing on myoblast proliferation, differentiation, and muscle protein synthesis signaling.

**Methods:**

Using human primary muscle cells, we examined the dose-response impact of flurbiprofen (25–200 µM), indomethacin (25–200 µM), ibuprofen (25–200 µM), and naproxen sodium (25–200 µM), on myoblast viability, myotube area, fusion, and prostaglandin production.

**Results:**

We found that supraphysiological concentrations of indomethacin inhibited myoblast proliferation (−74 ± 2% with 200 µM; −53 ± 3% with 100 µM; both *p* < 0.05) compared to control cells and impaired protein synthesis signaling pathways in myotubes, but only attenuated myotube fusion at the highest concentrations (−18 ± 2% with 200 µM, *p* < 0.05) compared to control myotubes. On the other hand, ibuprofen had no such effects. Naproxen sodium only increased cell proliferation at low concentrations (+36 ± 2% with 25 µM, *p* < 0.05), and flurbiprofen exhibited divergent impacts depending on the concentration whereby low concentrations improved cell proliferation (+17 ± 1% with 25 µM, *p* < 0.05) but high concentrations inhibited cell proliferation (−32 ± 1% with 200 µM, *p* < 0.05).

**Conclusion:**

Our findings suggest that indomethacin, at high concentrations, may detrimentally affect myoblast proliferation and differentiation via an AKT-dependent mechanism, and thus provide new understanding of NSAIDs’ effects on skeletal muscle cell development.

## 1. Introduction

In the United States, non-steroidal anti-inflammatory drugs (NSAIDs), such as ibuprofen, flurbiprofen, naproxen sodium, and indomethacin are widely used for their analgesic and anti-inflammatory properties [1–3]. According to surveys, 25% of female and 20% of male collegiate athletes use NSAIDs for pain relief or take them prophylactically [4]. Furthermore, more than 30% of recreationally active college students have reported using analgesics for exercise-related pain [5]. Military personnel use them at the highest rates, with more than 60% of Soldiers filling an NSAID prescription from 2006–2014 [6]. Taken together, NSAIDs are a popular choice for pain management and injury recovery in several populations.

NSAIDs act through inhibiting the enzyme cyclooxygenase 1 (COX-1), which is constitutively expressed, and/or cyclooxygenase 2 (COX-2), which is induced by exercise or injury and plays a role in pyrexia and pain [7]. The key distinction among NSAIDs lies in their affinity toward COX-1 and COX-2 enzymes, with their inhibitory actions being either preferential to one COX isoform (COX-1 or COX-2). Inhibition of COX-2 is typically associated with the beneficial anti-inflammatory, antipyretic, and analgesic effects of NSAIDs, whereas inhibiting COX-1 is largely responsible for their unwanted side effects, including gastrointestinal and renal toxicities [8]. The selectivity of an NSAID is measured by the inhibition concentration (IC) 50 or IC80 value, denoting the concentration required to inhibit 50% or 80% of COX activity, respectively [9] This is important in assessing the potential pharmacological effects of NSAIDs *in vivo* [10]. For example, indomethacin and flurbiprofen have a COX-1/COX-2 ratio of 10, while naproxen sodium has a COX-1/COX-2 ratio of 3.5 and ibuprofen has a ratio of 2.6 [11]. The differences in their affinity for COX enzymes accounts for differences among the NSAIDs on the secretion of prostaglandins, which play a role in muscle cell proliferation and fusion and are integral to musculoskeletal health (For review, see Trappe & Liu [1] and others [12,13]).

Previous research indicates that locally infusing 45 mg of indomethacin via microdialysis into the vastus lateralis of one leg over 7.5 hours interferes with satellite cells proliferation after eccentric exercise [14]. Others have found that 1,200 mg of ibuprofen in three 400-mg doses administered ∼30 minutes before and ∼6 hours and ∼12 hours following a bout of unaccustomed resistance exercise blunt muscle protein synthesis signaling [15]. Furthermore, ibuprofen (1,200 mg/day), but not acetaminophen (4,000 mg/day), inhibits protein synthesis in response to eccentric resistance exercise in humans [16]. In addition, NSAIDs such as celecoxib, have been reported to interfere with skeletal muscle growth and regeneration *in vitro* and *in vivo* [17–19]. However, the effects of other NSAIDs, such as indomethacin, flurbiprofen and naproxen sodium have seldom been studied in skeletal muscle, or in a dose-response manner in human primary muscle cells.

Therefore, given the high usage of NSAIDs among several populations and the importance of muscle cell proliferation and differentiation for skeletal muscle health, we investigated the effects of several NSAIDs on myoblast proliferation, differentiation, and intracellular signaling. We hypothesized that high concentrations (≥100 µM) of ibuprofen, naproxen sodium, indomethacin, and flurbiprofen would reduce myoblast proliferation and fusion but not low concentrations (≤50 µM).

## 2. Methods

### 2.1. Myoblasts and myotubes

Human Skeletal Myoblasts (Cat #2580), Skeletal Muscle Cell Growth Media and Bullet Kit (Cat #3245), Trypsin and Trypsin Neutralizing Solution Reagent Pack (Cat #5034) were obtained from Lonza Technologies (Portsmouth, NH, USA). Human Skeletal Myoblasts were grown and expanded in Skeletal Muscle Cell Growth Media and Bullet Kit at 37°C and 5% CO_2_. All experiments were performed within six passages of receipt from vendor (Lonza Technologies, Basel, Switzerland). Cells for proliferative experiments were grown and then seeded in six-well dishes for protein and 12-well dishes for immunofluorescence at 1.4 × 10^4^ cells/cm^2^. For differentiation experiments, the cells were seeded at 3.8 × 10^6^ cells/cm^2^ and then differentiated in low-glucose Dulbecco’s Modified Eagle’s Medium with 2% horse serum at 37°C and 5% CO_2_. Arachidonic acid (AA) was diluted in ethanol and cells were exposed, where appropriate, to various concentrations at a final concentration of 0.1%. In all cases, control cells were treated at equivalent concentration of Ethanol.

### 2.2. ELISAs

ELISA kits for PGE2 (Cat #514010) and PGF2α (Cat #516011) were purchased from Cayman Chemicals (Ann Arbor, MI, USA). Conditioned cell media was collected and prepared for ELISA analysis as previously described [18,20]. ELISAs were used to measure prostaglandin hormone concentrations PGE_2_ and PGF_2α_ from conditioned media.

### 2.3. Proliferation assays

The cell proliferation (#30-1010K) assay was purchased from ATCC (Manassas, VA, USA). Cell proliferation was measured using 3-(4,5-Dimethylthiazol-2-Yl)-2,5-Diphenyltetrazolium Bromide (MTT) assay. Human skeletal muscle myoblasts were allowed to grow for 48 hours and then the MTT assay was performed according to the manufacturer’s instructions.

### 2.4. Protein extraction and immunoblotting

Antibodies for Vinculin (#13901), p-p70 T389 (#9234), Total p70 (#2708), p-AKT S473 (#9271), Total AKT (#9272), p-S6 S235/236 (#4858), p-S6 S240/244 (2215), and Total S6 (#2217) were purchased from Cell Signaling Technologies (Danvers, MA, USA). Cellular protein extraction, Bradford Analysis and Immune Blotting were conducted as previously described [21,22]. Densitometric quantification analysis was done using NIH Image J 1.60 [23].

### 2.5. Immunocytochemistry, microscopy, and image analysis

Immunofluorescence antibody for myosin heavy chain (MyHc, #MF-20) was purchased from Developmental Studies Hybridoma Bank (Iowa City, IA, USA). Image J was downloaded from the National Institutes of Health website (Bethesda, MD, USA [23]). Cells were seeded and differentiated on collagen‐coated coverslips as previously described [20]. Images were captured using a Zeiss LSM700 confocal microscope and Zen analysis software. Results are presented as means ± standard deviation from independent experiments. Images were derived from five randomly captured fields for each treatment group. Myotube fusion index was determined by counting the nuclei in every myotube (defined as MyHC‐positive cells containing ≥ 2 nuclei) per field and dividing by the total number of nuclei in the field, as previously described [24].

### 2.6. Statistics

Data was checked for normality using a Kruskal-Wallis test, box plots, and Q-Q plots. Statistical analyses were performed using a one-way analysis of variance (ANOVA) with a Dunnett’s test post-hoc to compare each concentration with control condition if omnibus significance was found. A p-value of < 0.05 was considered significant. All measurements were repeated three to four times (*N* = 3–4 technical replicates).

## 3. Results

### 3.1. Flurbiprofen, indomethacin, and naproxen influence myoblast proliferation

**Figure 1.**
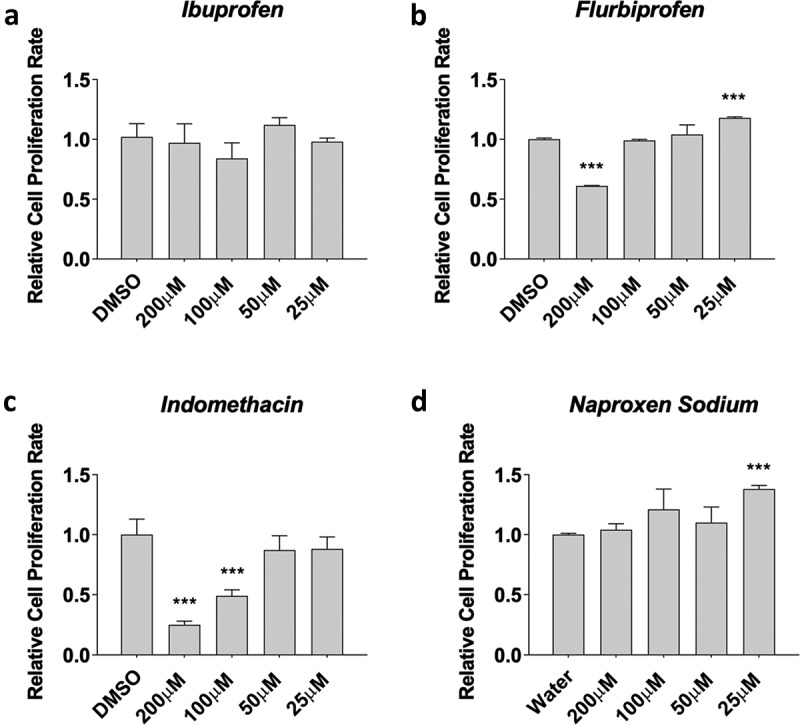
NSAIDs influence myoblast viability. Myoblasts were seeded and treated for a) ibuprofen, b) flurbiprofen, c), indomethacin, and d) naproxen sodium at various concentrations. MTT assay was used to assess cell viability. Data are expressed as mean ± SD from three independent experiments, each performed in duplicate (***p < 0.001; **p < 0.01, *p < 0.05).

First, we tested myoblasts treated with ibuprofen, flurbiprofen, indomethacin, and naproxen sodium for 48 hours during proliferation, which is a proxy of satellite cell proliferation in human muscle after injury or exercise. Data was normally distributed and statistical analyses were performed using an ANOVA with a Dunnett’s test post-hoc. We found no effects of ibuprofen on myoblast proliferation treated with 25 µM, 50 µM, 100 µM, and 200 µM concentrations (*p* > 0.05, Figure 1a). However, we found dichotomous results with flurbiprofen, where high concentrations (−32 ± 1% with 200 µM, *p* < 0.05) reduced proliferation and low concentrations (+17 ± 1% with 25 µM, *p* < 0.05) increased proliferation (Figure 1b) although 50 µM and 100 µM had no effect compared to control cells (*p* > 0.05). Indomethacin also reduced cell proliferation at high concentrations (−74 ± 2% with 200 µM; −53 ± 3% with 100 µM; both *p* < 0.05, Figure 1c) but not 50 µM and 100 µM concentrations compared to control cells. Naproxen sodium increased proliferation at low concentrations (+36 ± 2% with 25 µM, *p* < 0.05, Figure 1d) but had no effects at 50 µM, 100 µM and 200 µM concentrations compared to control cells.

### 3.2. Flurbiprofen, indomethacin, and naproxen influence myotube area and fusion

**Figure 2.**
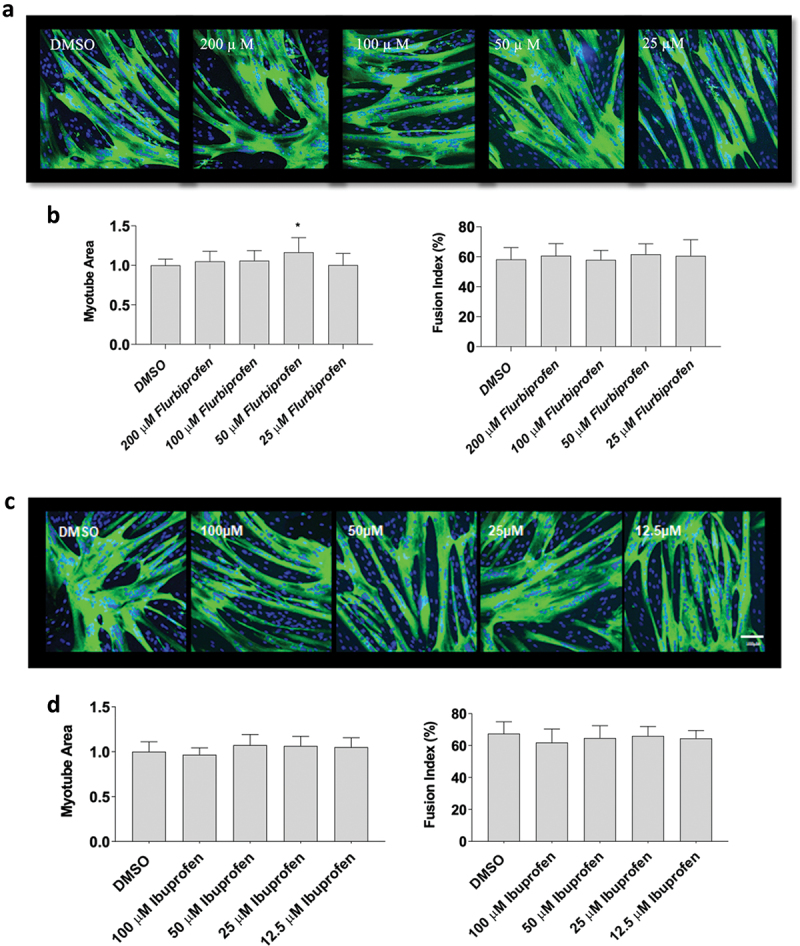
The effects of flurbiprofen and ibuprofen on myotube formation. Differentiating skeletal muscle myoblasts were treated with various concentrations of flurbiprofen or ibuprofen for 72 hours and then fixed and prepared for fluorescent confocal microscopy and gene using an antibody that recognizes embryonic myosin heavy chain. Representative images were generated using a Zeiss LSM 700 using Zen software. (a) representative images for flurbiprofen, (b) myotube area and myotube fusion percentage for flurbiprofen, (c) representative images for ibuprofen, and (d) myotube area and myotube fusion percentage for ibuprofen. Data are expressed as mean ± SD from three independent experiments, each performed in duplicate (***p < 0.001; ** *p* < 0.01, *p < 0.05).

**Figure 3.**
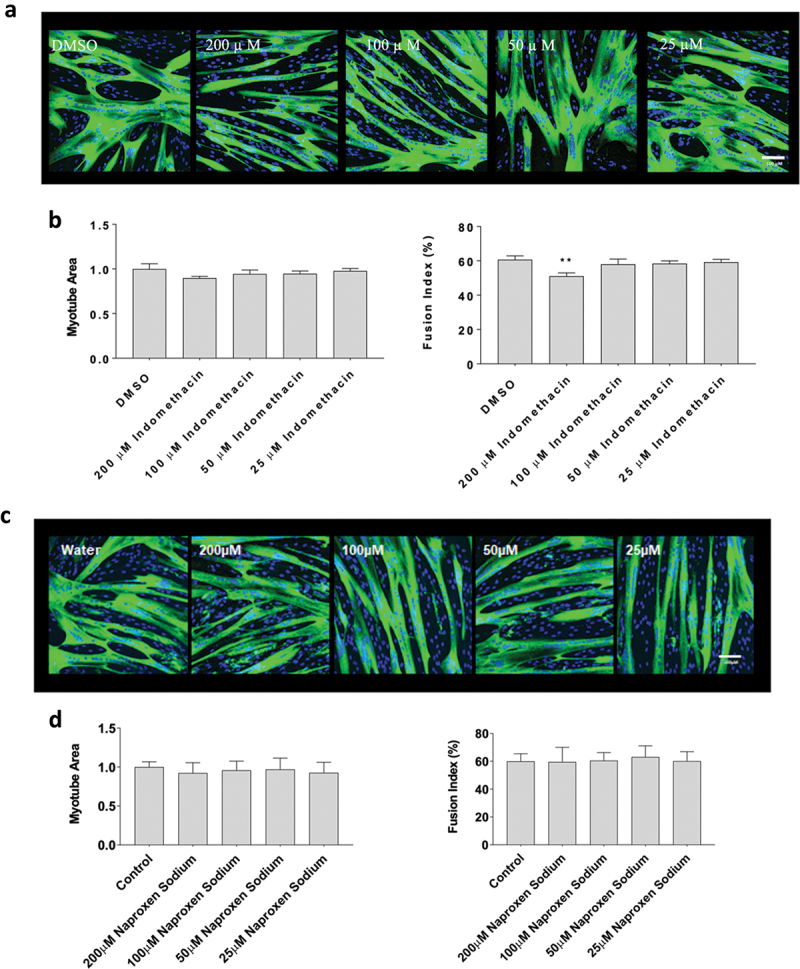
The effects of indomethacin and naproxen sodium on myotube formation. Differentiating skeletal muscle myoblasts were treated with various concentrations of flurbiprofen or ibuprofen for 72 hours and then fixed and prepared for fluorescent confocal microscopy and gene using an antibody that recognizes embryonic myosin heavy chain. Representative images were generated using a Zeiss LSM 700 using Zen software. (a) Representative images for indomethacin, (b) myotube area and myotube fusion percentage for indomethacin, (c) representative images for naproxen sodium, and (d) myotube area and myotube fusion percentage for naproxen sodium. Data are expressed as mean ± SD from three independent experiments, each performed in duplicate (***p < 0.001; **p < 0.01, *p < 0.05).

Next, since flurbiprofen, indomethacin, ibuprofen and naproxen had divergent effects on myoblast proliferation, we examined the influence of on changes in myotube area and fusion, which better recapitulates how NSAIDs may affect whole muscle changes in humans. Data did not violate assumptions of normality and statistical analyses were performed using an ANOVA with a Dunnett’s test post-hoc. Myotubes were exposed to flurbiprofen, indomethacin, or naproxen sodium at concentrations of 25 µM, 50 µM, 100 µM and 200 µM for 72-hours during differentiation. Flurbiprofen increased myotube area at 50 µM (+17 ± 1% with 25 µM, *p* < 0.05), but not at 25 µM, 100 µM, or 200 µM concentrations. Flurbiprofen also did not have any effects on fusion index at 25 µM, 50 µM, 100 µM and 200 µM (*p* > 0.05, Figure 2b). There were no effects of ibuprofen at any concentrations on myotube area or fusion index at any concentration, which falls in-line with the proliferation data (*p* > 0.05, Figure 2d). Representative images for flurbiprofen and ibuprofen can be seen in Figure 2a-c, respectively. Indomethacin reduced myotube fusion at high concentrations (−74 ± 2% with 200 µM; *p* < 0.01, Figure 3b,) but not at 25 µM, 50 µM, 100 µM concentrations (*p* > 0.05). Finally, there was no effect of naproxen sodium at 25 µM, 50 µM, 100 µM, or 200 µM concentrations on myotube area or fusion index (*p* > 0.05, Figure 3d). Representative images for indomethacin and naproxen can be seen in Figure 3a-c, respectively.

### 3.3. Indomethacin inhibits prostaglandin production in a dose dependent manner in myoblasts

**Figure 4.**
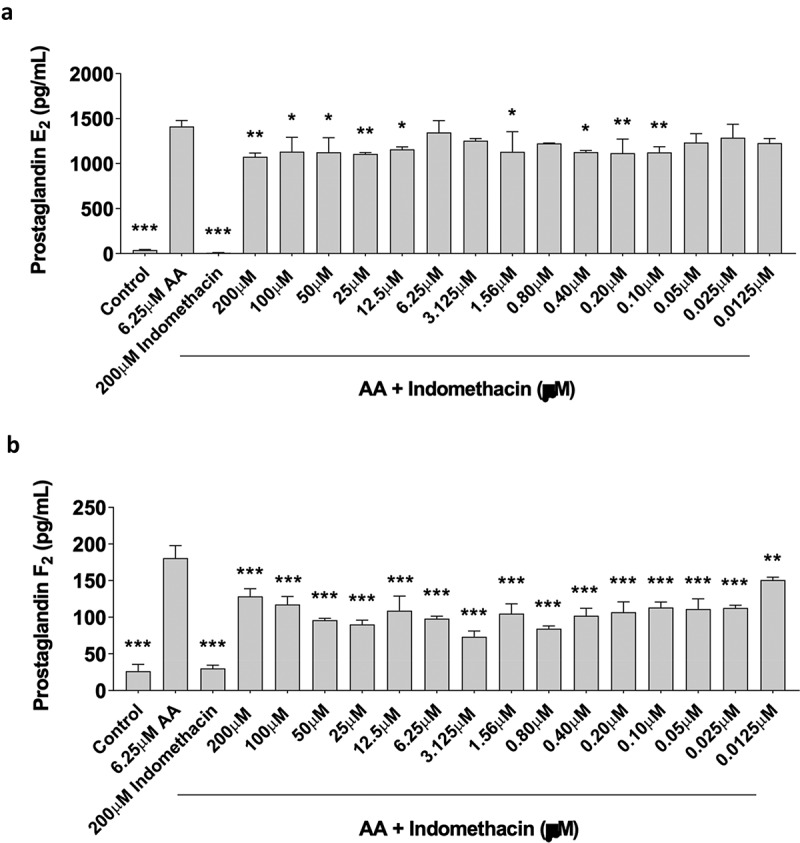
Indomethacin attenuates prostaglandin hormones. PGE_2_ and PGF_2α_ were measured using ELISAs. (a) PGE_2_ and (b) PGF_2α_ concentrations are shown for proliferating myoblasts treated with arachidonic acid and/or indomethacin for 72 hours. Data are expressed as mean ± SD (***p < 0.001; **p < 0.01; *p < 0.05; all compared to 6.25um AA).

Prostaglandin (PG) production is a common measure of COX activity [25]. The data exhibited a normal distribution, and we conducted statistical analyses utilizing an ANOVA, followed by a post-hoc Dunnett’s test. To determine the effects of indomethacin on PG synthesis, primary human skeletal myoblasts were treated for 48-hours with 6.25 µM arachidonic acid (AA) in the absence or presence of increasing concentrations of indomethacin (0.0125, 0.025, 0.05, 0.10, 0.20, 0.40, 0.80, 1.56, 3.125, 6.25, 12.5, 25, 50, 100, and 200 µM). We have previously shown that 6.25 µM AA can produce increases in PGE2 and PGF2 while improving myoblast proliferation and not interfering with myotube area or fusion – this model is meant to mimic the short-term muscle cell response that occurs post-exercise [20]. As expected, AA stimulated PGE2 and PGF2α production (Figure 4a, b). Furthermore, indomethacin reduced the AA-induced PGE2 response at 0.10, 0.20, 0.40, 0.80, 1.56, 12.5, 25, 50, 100, and 200 µM (*p* > 0.05) concentrations while the PGF2 response was reduced at all concentrations tested (0.0125–200 µM, *p* < 0.05).

### 3.4. Indomethacin impairs protein synthetic signaling pathways in primary human myotubes

**Figure 5.**
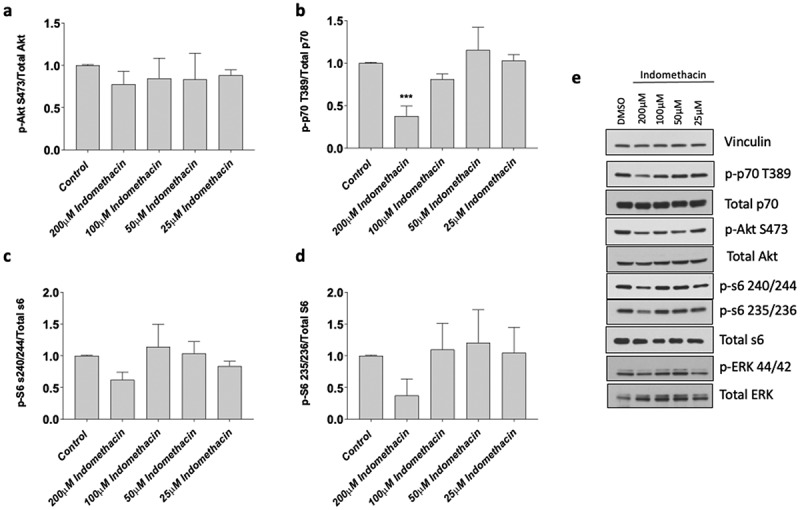
Effects of indomethacin on myogenic signaling. Myotubes were harvested after 72 h exposure to the indicated concentrations of indomethacin and protein was extracted. Western blotting was performed to evaluate the abundance of total and phosphorylated a) AKT (S473), b) p70S6K (T389), c) S6 (S235/236) and d) S6 (S240/244). Bands from phosphorylated p70S6K, AKT, and S6 were first normalized to total expression of these molecules, and then expressed relative to control, which was set at 1.0. Data are expressed as mean ± SD and represents 3 independent experiments (***p < 0.001; **p < 0.01; *p < 0.05).

Based on our myotube findings, we sought to characterize the effects of indomethacin on the muscle protein synthesis pathways that influence myoblast proliferation, myotube area and fusion at 25, 50, 100, and 200 µM concentrations. The data exhibited a normal distribution, and we conducted statistical analyses using an ANOVA followed by a Dunnett’s test as a post-hoc procedure. We found indomethacin at a concentration of 200 µM reduced the ratio of phospho-P70 to total p70 (−62 ± 4% with 200 µM, *p* < 0.001, Figure 5b) yet had no other effects on the AKT pathway although there were moderate nonsignificant reductions in the ratio of phosphor-S6 to total S6 at serine 235 and 473 (*p* > 0.05). This data indicates that high concentrations (200 µM) of indomethacin negatively influence muscle protein synthesis, which is likely the reason that saw reduced myotube fusion.

## 4. Discussion

In this study, we found that indomethacin inhibited proliferation of cultured primary human skeletal myoblasts at high concentrations (100–200 µM) but did not influence myotube size, and only attenuated myotube fusion at the highest concentration tested (200 µM). Furthermore, the 200 µM concentration of indomethacin reduced activation of signaling pathways related to protein synthesis and differentiation by 50–60% which likely contributed to the reduced fusion findings. These effects were not observed in cells treated with ibuprofen or naproxen sodium. However, concentrations of flurbiprofen had divergent effects on myoblasts where low concentrations improved cell proliferation (25 µM) while high concentrations reduced cell proliferation (200 µM). Taken together, our findings indicate that high concentrations of indomethacin, which are above blood plasma values found in humans taking indomethacin, negatively affect myoblast proliferation and differentiation of primary human skeletal muscle cells through an AKT-dependent mechanism.

Our study used a broad range of NSAIDs to understand which concentrations may be beneficial or detrimental to skeletal muscle. It is often pondered how *in vitro* drug experiments translate into concentrations seen in humans, thus context is provided for comparison. The circulating half-life of ibuprofen is three to four hours, flurbiprofen is three to six hours, indomethacin is five to ten hours, and naproxen sodium is twelve to eighteen hours [26–28]. These half-lives inform consumption and dictate levels found in human plasma. The NSAIDs administered in these experiments reach their peak concentration in blood plasma approximately two hours after ingestion and decline over time.

Naproxen sodium is generally taken in single dosages of 220 mg every 6–8 hours, with a maximum dosage of 660 mg per day. The concentrations found in human blood plasma for naproxen sodium on a 440 mg dose peak at 60 µg/mL and decays to 5 µg/mL by 72 hours [29]. This is comparable to our 200 µM concentration at the peak and a 25 µM concentration at 24 hours. Ibuprofen’s standard dosage for adults ranges from 200–800 mg every 6–8 hours, not exceeding 3,200 mg per day [30]. Participants who consumed 600 mg of ibuprofen three times per day, had a range of 43–63 µg/mL in their blood plasma [31] and more recent studies have found blood plasma concentrations with a single 600 mg dose range from 2–29 µg/mL [32]. Translating this to our experiments, it would represent the ~200 µm of ibuprofen at its peak and ~12.5 µm at the lowest dose found in humans. Flurbiprofen is generally taken in dosages of 50 mg every four to six hours, with a maximum of 200 mg per day in most cases [33,34]. In human plasma, 100 mg causes flurbiprofen blood plasma to peak at ~45 µg/mL after two hours and decays to ~10 µg/mL eight hours later [35], which is comparable to our 200 µm and 50 µm concentrations in cells, respectively. Cumulatively, our NSAID experiments broadly recapitulated concentrations seen in humans.

Indomethacin is typically taken at 25 mg three times a day or 40–50 mg two to three times a day with a maximum dose of 200 mg per day [36,37]. In humans, a 25 mg oral dose produces peak indomethacin concentrations of ~2.5 µg/mL in blood plasma by ~2.5 hours, and decays to 0.2 µg/mL by 8 hours. The peak human concentration on a 50 mg dose is comparable to a ~12.5 µm concentration *in vitro*. Indomethacin is a COX inhibitor with a 10-fold higher affinity for COX-1 compared to COX-2 enzymes, that has been used to study muscle growth and regeneration. For example, myogenesis was inhibited in C2C12 muscle cells treated with 100 or 200 µM of indomethacin [38]. Our results concur with and expand on these findings. By testing lower concentrations of indomethacin, which are closer, but still double the concentrations found in human plasma, we observed no alterations in myogenesis at 25 µM. Therefore, given these high concentrations it is unlikely they influence muscle cells in humans who consume low or moderate dose NSAIDs due to general pain associated with unaccustomed or intense exercise. However, it is also important to remember that NSAIDs are usually not taken unless pain or inflammation is present, but they may still influence healthy skeletal muscle outside of the affected areas (e.g. affect biceps when being taken for a hamstring strain), and our results suggest indomethacin may reduce muscle proliferation only at the highest concentrations. This may make it a viable alternative to ibuprofen and acetaminophen, which have negative effects on muscle protein synthesis post exercise [1,14,39]. However, there is a trade-off of potential gastrointestinal or renal concerns with indomethacin if taken at high dosages for long periods [40].

Arachidonic acid is often used to mimic exercise or inflammation *in vivo* [20,41,42]. Our current experiments suggest that 6.25 µM AA induces PGE_2_ and PGF_2α_, which is in-line with previous work [20]. When treated with varying concentrations of indomethacin, the elevation in PGE_2_ was reduced in most conditions, except those with extremely low concentrations of indomethacin. PGF_2α_ production was also reduced when cells were treated with varying concentrations of indomethacin although neither prostaglandin elicited a dose-response effect. The only concentration that completely attenuated the prostaglandin response was 200 µM of indomethacin, which is likely the reason we saw reductions in myoblast proliferation and myotube phenotype at those concentrations.

The COX pathway plays a vital role in controlling satellite cells, as demonstrated by various studies, and can be studied using *in vitro* methods [39,43–46]. Specifically, when COX-2 is inhibited, the proliferation of satellite cells decreases. For example, combined inhibition of COX-1 and COX-2 leads to reduced myotube differentiation and fusion of satellite cells [43]. While previous studies did not use indomethacin, their findings align with ours, indicating that high concentrations of COX-1 and COX-2 inhibitors can disrupt myoblast proliferation and myotube fusion [43]. More directly related to our research, studies that have employed indomethacin to suppress prostaglandin synthesis also reported a decrease in myotube fusion [47], which aligns closely with our observations, further solidifying the role of the COX-1 pathway in muscle cell homeostasis.

Our study also highlights some interesting findings with ibuprofen, flurbiprofen, and naproxen sodium. Previous research has indicated that ibuprofen inhibits the muscle protein synthesis (MPS) response after exercise in humans, but not proliferation in muscle cells [16,48]. Other data suggests COX-2 activity is required for skeletal muscle hypertrophy and cell proliferation [49–51] and helps maintain protein turnover [52]. On the other hand, it appears that 45 mg of indomethacin does not inhibit the MPS response [53]. In combination with our findings, this could be interpreted as indomethacin being a better choice for athletes recovering from injury or exercise related to skeletal muscle.

To our knowledge, only three studies have identified the effects of flurbiprofen on skeletal muscle. In the first, Mishra et al., found that 9 mg of flurbiprofen induced a short-term gain in muscle function, but then a subsequent functional reduction in rabbits who underwent exercise-induced injury [54]. In the second study, Semark et al., found that 40 mg of flurbiprofen administered as patches before and after exercise caused no reductions in delayed-onset muscle soreness, measured by both a pressure probe and by subjective assessment, in rugby players who completed an exercise protocol to induce muscle damage [55]. Lastly, in a study comparing a 150 mg dose of flurbiprofen to 3,600 mg dose of aspirin, a study found that flurbiprofen was significantly better in reducing the time taken to achieve both training and match fitness in professional football players, and > 60% of the players taking flurbiprofen were able to train within three days of injury compared to > 30% taking aspirin [56]. We found that flurbiprofen may have a dose-dependent effect because low concentrations improve myoblast proliferation but high concentrations reduced it; however, given the equivocal findings in the literature this is difficult to translate to recommendations for humans. Thus, more research is needed to better understand if and/or when flurbiprofen could be beneficial or detrimental to skeletal muscle especially regarding injuries or performance. Interestingly, when comparing the flurbiprofen to indomethacin results in our experiments, where both have a 10-fold more affinity for COX-1 than COX-2, the mechanism underlying the proliferation findings of flurbiprofen are likely not driven via COX-1 and could be off-target effects.

Our study has strengths and limitations. A notable advantage of our study was the implementation of a dose-response approach, which facilitated a systematic examination of the effects of flurbiprofen, ibuprofen, indomethacin, and naproxen sodium on proliferating and differentiating myoblasts. Additionally, there are many regulatory and metabolic redundant pathways that we unfortunately cannot mimic using cultures. The NSAID concentrations used in our experiments are similar to those seen in humans for ibuprofen, flurbiprofen, naproxen sodium, but we cannot rule out that they could be different than those found specifically around the muscle. There is some evidence that suggests levels in blood and other tissues are similar for indomethacin [57]. Another factor that plays a role in NSAID blood plasma concentrations are nutrient intake, with slightly higher concentrations found in humans who are fasting [58]. Our cells were not fasted and were given ample resources in the media to proliferate, fuse, and grow alongside NSAID treatment. Future work could develop models that replicate the human response to muscle injury to better identify NSAID effects. A strength of our study is that we conducted experiments at various stages of the myogenic process, spanning from myoblast proliferation to terminal differentiation, thereby providing a broad overview of processes within a single cell type. We also identified a mechanism through which indomethacin may act to reduce myoblast proliferation and differentiation; however, it is unlikely that we identified all processes or pathways that contribute to these reductions. There is also a potential for off-target effects when using high concentrations of any drug, which could contribute to our findings.

In conclusion, we found that high concentrations of indomethacin (200 µM) inhibited myoblast proliferation and impaired protein synthesis signaling pathways in myotubes; however, ibuprofen or naproxen displayed no effects in myoblasts or myotubes, and flurbiprofen increased myoblast proliferation at low concentrations (50 µM) and decreased proliferation at high concentrations (200 µM). Collectively, our experimental data utilizing ibuprofen, flurbiprofen, and naproxen contribute to the existing body of literature, offering novel insights and enriching the ongoing scientific discussion. Future experiments should test NSAIDs in a translational fashion to determine their precise impacts on muscle recovery, repair, and growth following different types and intensities of exercise, while also considering potential long-term effects and the role of individual genetic, dietary, and lifestyle factors. This will help optimize the use of these medications in sports medicine and physical rehabilitation and contribute to appropriate treatment strategies.
